# Supplementary material for: Indigenous Voices Against Suicide: A Meta-Synthesis Advancing Prevention Strategies
Source: Int J Environ Res Public Health. 2023 Nov 15;20(22):7064. doi: 10.3390/ijerph20227064 (PMC10671466; doi:10.3390/ijerph20227064)
Supplement: Supplementary file 1 [file ijerph-20-07064-s001.zip › ijerph-2577661-Supplementary Materials.pdf]

Supplementary Materials Table S1. Summary of suicide prevention interventions for Indigenous Peoples and corresponding themes (N = 15).

Authors	Sample size	Population	Subpopulation	Program	Methodology	Q1 Category Code(s)	Themes
Antonio et al. (2020)	N=26	Native Hawaiian<br>Hawaii<br>Pacific Islander	YYA<br>(13-18 years)<br>Rural	Hawaiʻi's Caring Communities Initiative (HCCI) for Youth Suicide Prevention	Qualitative Methods<br>Semi-structured interviews<br>Focus groups	1, 2	b
Burrage et al. (2016)	N=15	American Indian<br>Midwest	YYA + Adults<br>(13-79 years)<br>Urban	Midwestern Urban Indian Health Organization (UIHO) Community Readiness Assessment (CRA) Urban AI Youth suicide project	Demographics<br>Semi-structured interviews<br>PAR	1, 3	a, b, c
Cwik et al. (2019)	N=112	American Indian<br>Fort Apache Reservation in east central Arizona	YYA<br>(Ages not specified, 7th and 8th grade)<br>Tribal	Apache Celebrating Life (CL) Apache Elders' Prevention Curriculum Pilot	Elders Council meetings and minimally structured qualitative interviews<br>CBPR	3, 4	b, c

DeCou et al. (2013)	N=25	Alaska Native	University Students<br>Adults<br>(18-37 years)<br>Rural	Alaska Native college students' discussion and beliefsof suicide<br>Alaska Native Health Research (CANHR)	Qualitative<br>Semi-structured interviews	1	a, c, d
de Schweinitz et al. (2017)	N=54	Alaska Native Athabascan Native Villages Interior Alaska	YYA<br>(Ages not specified)<br>Rural	Village Wellness Team Program – youth suicide prevention	Qualitative<br>Focus Groups<br>CBPR	2, 4	a, c, d
Freedenthal & Stiffman (2007)	N=101	American Indian<br>Southwest	YYA<br>(15-21 years)<br>Urban<br>Tribal<br>Reservation	American Indian Multisector Help Inquiry (AIM-HI)<br>Year 3 subsample	Mixed Methods	1	a, b
Holliday et al. (2018)	N=148	American Indian<br>Suquamish Tribe<br>Port Gamble Tribe<br>PNW<br>Washington State	enrolled members<br>Elders<br>Youth<br>(14-20 years)<br>Tribal	Community assessment to inform a pilot study on suicide and substance use	Mixed Methods<br>Semi-structured interviews<br>Photovoice<br>Digital Storytelling<br>CBPR	1, 2, 3, 4	a, c
Jansen et al. (2021)	N=189	American Indian<br>Alaska Native	Adult<br>(18+ years)<br>Urban<br>Tribal	Caring Contacts suicide prevention intervention	Qualitative<br>Focus groups<br>Semi-structured interviews<br>CBPR	1, 2, 3, 4	b, c
Le & Gobert (2015)	N=8	CSKT of the Flathead Reservation<br>Montana	Youth<br>(15-20 years)<br>Tribal<br>Rural	Mind Body Awareness Project (MBA)<br>Mindfulness-Based Stress Reduction program (MBSR)<br>Pilot study	Mixed Methods<br>Open-ended interviews	1, 3, 4	d

Rasmus (2014)	N=33	Alaska Native<br>Yup'ik<br>Southwest Alaska	Youth<br>Adult<br>Elders<br>Rural<br>Village	Elluam Tungiinun (Towards Wellness) Program Evaluation	Qualitative<br>Evaluation<br>Focus groups<br>Semi-structured interviews<br>CBPR	1, 2, 3, 4	c, d
Rasmus et al. (2014)	Individuals not identified N=3 (case studies)	Alaska Native<br>Yup'ik<br>Southwest Alaska	Youth<br>Adults<br>Rural<br>Village	Qungasvik (A Yup'ik Intervention ''Toolbox'')	Qualitative<br>Community-driven development of a cultural prevention program<br>CBPR	3, 4	a, b, c
Shaw et al. (2019)	N=15	American Indian<br>Alaska Native<br>Anchorage, AK	YYA<br>Adults<br>(15-56 years)<br>Rural<br>Tribal<br>Reservation	Southcentral Foundation (SCF) Behavioral Urgent Response Team (BURT)	Qualitative<br>Methods interpretive phenomenologic al analysis<br>(IPA)	1	a, b
Skewes et al. 2022	N=25	Alaska Native	YYA<br>(18-37)<br>Rural	Gain insight regarding suicide from Alaska Native university students from rural villages that moved to urban areas for education	Qualitative<br>Semi-structured interviews	1	b, c, d
Strickland et al. (2006)	N=49	American Indian<br>Pacific Northwest Tribe	Adults<br>Parents<br>Elders<br>Tribal	A suicide risk assessment program	Qualitative<br>Focus groups<br>Semi-structured interviews	1, 2, 3	b, c
Trout et al. (2018)	Individuals not identified	Alaska Native	Not identified	Promoting Community	Qualitative<br>Learning circles	2	a, b, c

	N=10 (villages)	Northwest Alaska		Conversations About Research to End Suicide (PC CARES)	Decoloniality Framework		

Note. Q1 category codes are as follows: 1) interviews were conducted with participants; 2) focus groups, learning labs or equivalent were conducted with participants; 3) a community advisory board, group, or committee was included; 4) inclusion of tribal leadership (if tribal-specific); 5) authors explicitly note how participant perspectives were included although none of the prior categories were found in the article; or (6) no indication of the engagement of participant perspectives informing program development was outlined. Themes are coded as: (a) support preferences; (b) challenges to suicide programming; (c) integration of culture as prevention, and (d) grounding relationship in prevention.

Supplementary Materials. Sources and Search Protocol

**Population Descriptors**	**Content Terms**
American Indian OR Alaska Native OR American Indian and Alaska Native OR AI/AN OR Native American OR Native Hawaiian	Suicide Intervention OR Suicide Prevention OR Suicide Program

Figure 1: Search term combinations as outlined by population descriptors and content terms.

PubMed

Advanced Search
'Show index' - 'AND' selected
Search string - Population AND Content
EX: "American Indian and Alaska Native" AND "Suicide Intervention"
Text Availability – check 'Abstract' and 'Full Text'
Display Option – Format 'Abstract' – sort by 'best-match'- set 200 searches per page for better viewing
Save – Selection 'All results' – Format 'PubMed' – click 'create file' and save
EX: '(1) AIAN…' – indicating 'American Indian and Alaska Native' content term 'Suicide Intervention'
Import PubMed txt file to Zotero into separate folder for easy reference of specific search string pull

Search String Initial Counts

(1) AIAN - 21
(2) AIAN - 26
(3) AIAN - 12
(1) AK - 110
(2) AK - 122
(3) AK – 59
(1) AI – 147
(2) AI – 165
(3) AI – 82
(1) AIandAN – 64
(2) AIandAN – 73
(3) AIandAN – 35
(1) NA – 139
(2) NA – 159
(3) NA – 80
(1) NH – 78
(2) NH – 76
(3) NH – 45

Initial Pull Count – 1493

PsychInfo

Advanced Search

Search string - Population AND Content

EX: "American Indian and Alaska Native" AND "Suicide Intervention"

'Limit to' – check 'peer reviewed'
Toward bottom of search outcome page make sure viewing of results is 100 per page
Leave search 'sorted by' based on 'relevance'
Select all search results per box – if more than 100, select box of results per page for each search

EX: 'Select 1-20' – check box

Click to the right of the box the "…" circle in blue indicating 'All save options'
Click on "RIS" format icon
Then 'deselect items when done' – check box as to not carry over unnecessary created duplicates
Download RIS txt file

EX: '(1) AIAN…'

Import PsychInfo txt file into Zotero into separate folder for easy reference of specific search string pull

Search String Initial Counts

(1) AIAN – 20
(2) AIAN – 23
(3) AIAN – 17
(1) AK – 65
(2) AK – 92
(3) AK – 49
(1) AI – 97
(2) AI – 165
(3) AI – 101
(1) AIandAK – 57
(2) AIandAK – 83
(3) AIandAK – 46
(1) NA – 89
(2) NA – 123
(3) NA – 75
(1) NH – 6
(2) NH – 9
(3) NH – 9

Initial Pull Count – 1126

SAGE Journals

Advanced Search
Under 'Publication' check – 'All dates'
Access Type – check 'Only content to which I have full access'
Type in search string as advanced search allows connection of Boolean operator 'AND' – click '+ Add search'
Search string - Population AND Content

EX: "American Indian and Alaska Native" AND "Suicide Intervention"

Keep as 'Full Text'
Results per page '100' and maintain sort by 'relevance' – 'Article type' – 'Research Article' only

Go to each search results page and select all
Check 'Select all' – click on 'Download select citations'
Download RIS txt file
    EX: '(1) AIAN…'
Then uncheck 'select all' – before entering new search as to not carry over unnecessary created duplicates
Import SAGE txt file into Zotero into separate folder for easy reference of specific search string pull

Search String Initial Counts

(1) AIAN – 9
(2) AIAN – 8
(3) AIAN – 7
(1) AK – 78
(2) AK – 86
(3) AK – 91
(1) AI – 360
(2) AI – 396
(3) AI – 380
(1) AIandAK – 66
(2) AIandAK – 69
(3) AIandAK – 75
(1) NA – 342
(2) NA – 370
(3) NA – 376
(1) NH – 48
(2) NH – 48
(3) NH – 54

Initial Pull Count – 2863

TOTAL Initial Count – 5482

Merge duplicated items into one item in Zotero under 'Duplicate Items' - 1438

Non-full content removed - 52
Booklets & brochures (1), letter (2), annual meetings (6), indices (42), abstract lists (3)

Unduplicated Count and non-full content removed– 1386

Title and Abstract Screening

Filters 1 and 2 can be done interchangeably during the same initial screening. Referencing the title and the abstract in the right had panel under 'Info' tab located at the top of the panel.

Filter 1 will screen titles for relevance, which will be defined as the reporting of suicidality studies, interventions or programs implemented exclusively with Indigenous Peoples in the United States. These programs or interventions can include other outcomes as well such as substance misuse, depression, anxiety, etc.

Filter 2 will involve the review of abstracts of all preliminarily screened titles for the inclusion of qualitative methodologies, whether mixed-methods or exclusively qualitative in nature of a study, program or intervention.

Sign into WSU Libraries SEARCH IT - https://searchit.libraries.wsu.edu/primo-explore/search?vid=WSU&lang=en_US&sortby=rank

During title and abstract screening within Zotero – if it seems to be unclear, or if title seems relevant, but abstract is limited or brief, a review of the full article may be needed to determine inclusion or exclusion:

> For example, if a title or abstract references psychological distress or behavioral health outcomes generally, a brief review of the article is needed to scan for suicide relevance via the 'control-F' function. Again, suicide would need to be an outcome, not solely referenced – let's say in the background or introduction sections this can be common.

It may be that in the title and abstract it is still unclear at your initial read, whether Indigenous Peoples are the sole population. You may also need to undergo this step if the title and abstract does not specifically indicate that the article is in reference to an intervention or program – at times the article may be contextual in nature, a systematic review, surveillance reporting, literature reviews, secondary analyses, or epidemiological (these will not be included and should not be tagged). Note, that if suicidality is an outcome, it can be included within this filter.

In addition, as to whether the intervention utilized qualitative methods, it may also be that a full article access is not available to confirm inclusion. In this case – note 'F12-1(or 2) P' for 'pending'. Once the articles come in through Inter-library loan and reviewed the tag can be changed to 'F12-1 (or 2)' or the tag removed completely.

If confirmed based on the criterion noted above, click the 'Tag' tab located at the top of the panel.
Tag in Zotero if title and abstract hit all the criteria above and then tag as 'F12-1' or 'F12-2' for reviewer 1 and 2.

Upon review and consensus of articles that underwent Filters 1-2 to assess the following: Titles, authors, abstracts, and full text publications, to track inclusion and exclusion decisions prior to moving onto. Those articles tagged as F12-C underwent conferencing between conferencing to assess the articles that underwent F3.

Filter 3 for final determination of the sample that will be accompanied with synthesis memos.

F12-1 = 24
F12-2 = 24

Conference Meeting March 11th, 2022

F12-C = 14
F-3 = 20

F3=24

The 9 articles remaining upon title and abstract screenings will undergo Filter 3, a full article review tagged as F3. Within this filter the inclusion of action research will be assessed by the inclusion of words and phrases that embody the approach such as, ‘community-based participatory research (CBPR)’, ‘participatory action research (PAR)’, ‘community-based’, ‘community-engaged’, ‘decolonization’, ‘decolonizing’, action ‘transformative-emancipatory perspective’, ‘action research’, etc.

Conference Meeting March 31st, 2022

Confirmed FINAL SAMPLE = 15

Supplementary Materials. Codebook

Primary Code
Secondary Code(s)

Resiliency - The strength, persistence and balance of a peoples who have endured many stressors and barriers to have continued to share and be amidst a community of knowledge, culture and well-being. Strengths-based livelihoods and connections that foster empowerment, collective thriving, and generational security in identities and values systems.

Oral Tradition – The practice of oral storytelling and knowledge sharing, removed from the written word.

Intergenerational Knowledge – The sharing of knowledge (e.g., traditions, culture, language) through intergenerational exchange, from elders, community members, youth – the cyclical nature of sharing understanding from a multi-generational perspective.

Cultural Activities as Protective Factors – Cultural and traditional activities as protective factors as healing and preventative practices.

Cultural Identity - The expression or explanation of identity or intersectionality of identity with ones tribal and/or Indigenous cultural connection and ties.

Kinship – The connection and reciprocal ties between family systems, community and nature, whether it be connection to other human relatives through family systems, and naming practices or relationship with our other plant, sky, water and land relatives. The understanding and knowledge systems that center the connection to all things, humans as not apart from all else. Highlighting communities of care and collaboration.

Family Systems – Understanding of the family system as non-nuclear, rather supporting from a community caregiving approach. Influences of the caregiver-child relationship on emotion regulation, social dynamics and overall health and wellness. Focus on naming ceremonies and ways of introducing oneself and noting who you are, where you come from as contributing to a sense of cultural identity. Any reference to family dynamics strengths or barriers.

Historical Trauma – The discussion or reference to the impact of intergenerational trauma due to settler-colonialism (e.g., boarding schools, geographical relocation, loss of culture, perpetuation of stereotypes).

Confounding Risk Factors - The note of factors that contribute to the risk for suicide as brought about from historical trauma (e.g., substance use, economy, employment

Service Utility – Discussion and sharing surrounding access to health and human services to support prevention and treatment of suicidality as well as confounding needs that may impact ideation and risk.

Informal Supports - The availability of safe space and crisis or prevention efforts through social and behavioral health support from paraprofessionals, family, community members, mentors, or friends.

Formal Supports – The availability of formal services from licensed or experiences professionals such as mental therapists, counselors, doctors, nurses and other healthcare providers and inpatient or outpatient treatments.

Barriers to Care and Utility – The description or discussion of challenges, barriers, stigma, and/or fear in accessing support systems or care services as well as the use of these services in each community respectively.

Awareness – The state of knowledge or education of suicidality awareness in the community. References to the limit or expansion of this awareness.

Community Informed Programming

Advisory Bodies – The integration and foundation in the inclusion of advisory committee, community advisory boards inclusive of elders, leaders, community members, etc. as decision makers and guidance for programming and cultural teachings.

Tribal Leadership – The notation of partnership, approval and guidance provided from a sovereign nations' tribal council or governing bodies for an Indigenous community.

Integration of Cultural Teachings and language - Focus on integration and access to cultural healing, language practices and traditional medicine practices for prevention and treatment.

Data Sovereignty – Community and tribal ownership of the program strategies, data and dissemination of materials and/or products.

Academic Partnerships - Identifying university or academic partnerships with Indigenous community(ies) in research and program development, implementation, dissemination and/or sustainability efforts.

YYA – Programming and approach targeted for Youth and Young Adults, focus on younger generations for prevention or intervention efforts.

Adults – Programming and approach targeted for adults generally, focus on prevention broadly, adulthood-elderhood.

Tribal – Programming and approach focused on sovereign nations and tribal – rural communities, reservation or village environments.

Urban – Programming and approach focused on urban Indian communities.
